# Gray Matter Hypoxia in the Brain of the Experimental Autoimmune Encephalomyelitis Model of Multiple Sclerosis

**DOI:** 10.1371/journal.pone.0167196

**Published:** 2016-12-01

**Authors:** Thomas W. Johnson, Ying Wu, Nabeela Nathoo, James A. Rogers, V. Wee Yong, Jeff F. Dunn

**Affiliations:** 1 Department of Radiology, Cumming School of Medicine, University of Calgary, Calgary, Alberta, Canada; 2 Department of Clinical Neurosciences, Cumming School of Medicine, University of Calgary, Calgary, Alberta, Canada; Hopital Robert Debre, FRANCE

## Abstract

**Background:**

Multiple sclerosis (MS) has a significant inflammatory component and may have significant gray matter (GM) pathophysiology. Brain oxygenation is a sensitive measurement of the balance between metabolic need and oxygen delivery. There is evidence that inflammation and hypoxia are interdependent. In this paper, we applied novel, implanted PO_2_ sensors to measure hypoxia in cortical and cerebellar GM, in an inflammation-induced mouse model of MS.

**Objective:**

Quantify oxygenation in cortical and cerebellar GM in the awake, unrestrained experimental autoimmune encephalomyelitis (EAE) mouse model and to relate the results to symptom level and disease time-course.

**Methods:**

C57BL/6 mice were implanted with a fiber-optic sensor in the cerebellum (n = 13) and cortex (n = 24). Animals were induced with stimulation of the immune response and sensitization to myelin oligodendrocyte glycoprotein (MOG). Controls did not have MOG. We measured PO_2_ in awake, unrestrained animals from pre-induction (baseline) up to 36 days post-induction for EAE and controls.

**Results:**

There were more days with hypoxia than hyperoxia (cerebellum: 34/67 vs. 18/67 days; cortex: 85/112 vs. 22/112) compared to time-matched controls. The average decline in PO_2_ on days that were significantly lower than time-matched controls was -8.8±6.0 mmHg (mean ± SD) for the cerebellum and -8.0±4.6 for the cortex. Conversely, the average increase in PO_2_ on days that were significantly hyperoxic was +3.2±2.8 mmHg (mean ± SD) for the cerebellum and +0.8±2.1 for the cortex. Cortical hypoxia related to increased behavioral deficits. Evidence for hypoxia occurred before measurable behavioral deficits.

**Conclusions:**

A highly inflammatory condition primed to a white matter (WM) autoimmune response correlates with significant hypoxia and increased variation in oxygenation in GM of both cerebellum and cortex in the mouse EAE model of MS.

## Introduction

Multiple sclerosis (MS) is an immune-mediated [1, 2], neurodegenerative disorder. Although there is uncertainty about the initial trigger for MS, almost all patients will face an irreversible progressive form of the disease which leads to neurological deterioration [3]. The ultimate cause of both MS and the progressive neurodegeneration is still largely unknown [4].

Although MS is historically considered a white matter (WM) disease, there is strong evidence for diffuse gray matter (GM) involvement [5]. For example, atrophy of brain GM is greater in patients that develop MS than those who do not [6] and predicts the conversion from clinically isolated syndrome to definite MS [7]. In addition, GM atrophy accelerates as the disease progresses, whereas WM atrophy remains constant [8]. GM atrophy correlates with both cognitive [9–11] and physical disability [8, 12] and more accurately predicts some of these disabilities than WM changes [7, 13]. Furthermore, GM atrophy has been observed in the whole brain, cerebral cortex [14] and cerebellum [15, 16] of experimental autoimmune encephalomyelitis (EAE) mice, a well-established animal model of MS; this atrophy was found to correlate with disease load [14]. These lines of evidence indicate that GM degeneration is a driver of disability in progressive stages of the disease [5] and the EAE model may be useful in studying its pathophysiology.

An important link exists between neurodegeneration and brain metabolism that was illustrated in a study by Brooks *et al*.: using positron emission tomography (PET) in progressive MS patients, they observed reduced cerebral metabolic rate of oxygen metabolism (CMRO_2_), which correlated with a reduction in GM volume and cognitive deficits [17]. It is also known that changes in brain metabolism, inflammation and tissue oxygenation are interrelated and can exacerbate one another [18], with strong evidence of an interaction between hypoxia and inflammation. An intracellular environment low in oxygen may result in the upregulation of the master hypoxia response regulator, hypoxia-inducible factor 1α (HIF-1α) [19], that can cause upregulation and activation of the immune modulator, ‘nuclear factor kappa-light-chain-enhancer of activated B cells’ (NF-kB). Thus, if hypoxia is present in a tissue, it could be indirect evidence of inflammation or a modulator of the inflammation itself. This is particularly relevant to MS due to the strong inflammatory component of the disease. Indeed, histological evidence exists for hypoxia in MS, including: increased endoplasmic reticulum (ER) stress and hypoxia-associated molecules in GM lesions [20], as well as hypoxia-like WM lesions [21] that show increased HIF-1α [22] and its downstream signaling molecules, such as vascular endothelial growth factor (VEGF) and glucose transporter 3 (GLUT-3) [23].

Quantitative near-infrared spectroscopy data indicates that as many as half of MS patients may have hypoxia in the cortex [24]. In EAE mice, MRI measurements sensitive to deoxyhemoglobin provided evidence for hypoxia in the spinal cord [25]. In addition, low PO_2_ (hypoxia) has been detected in spinal cord of a rat EAE model while under anesthesia [26] and was found to promote demyelination in the lipopolysaccharide model of MS lesions [27].

We hypothesized that we would detect hypoxia in cortical and cerebellar GM of EAE mice. To minimize the impact of animal restraint and anesthesia on PO_2_ values, we used a novel chronically implanted sensor that allowed for repeated measurement in awake, unrestrained animals [28].

This paper provides new evidence supporting our hypothesis. We also report increased variability of oxygenation, with some days that are hyperoxic. Finally, we report that tissue hypoxia appears to occur later in the cortex than in the cerebellum.

## Methods

Female C57BL6 mice (Charles River Laboratories, 8–10 weeks) were housed with a 12-hour light and dark cycle. Food and water were available *ad libitum*. Protocols were approved by the University of Calgary Animal Care Committee and meet the Canadian Council of Animal Care guidelines.

### Study Design

PO_2_ probes were implanted one week after mouse arrival and structural MRI was obtained 1–2 days after surgery to confirm the location of the PO_2_ probe. Beginning 3–6 days after surgery, baseline PO_2_ measurements were taken for 10 minutes per day at a sampling rate of 0.1Hz (60 data points total) on 3 consecutive days in awake mice. EAE induction was then performed the day after the final baseline measurement. Before and during disease peak (days 10–18 post-induction), measurement frequency was once every 2–3 days and decreased to once to every 5 days after the peak period. Measurements were preceded by a 15-minute acclimation period and were collected during the same 4-hour period in the afternoon for all animals. Most animals were sacrificed between days 28–36 post-induction, at which time brain tissue was perfused and fixed.

### Humane Endpoint

Animal behavior was monitored every 3–5 days and every other day during the disease peak (days 10–18). Animals were humanely sacrificed if their behavior deficit was 13/15 or greater (see below for behavior measurement description) or if there were surgical complications, such as a loose probe implant. In addition, mice were inspected daily to determine if their mobility was deteriorating and were humanely sacrificed if they were unable to feed themselves. This was simple to track as each mouse was housed in a separate cage and thus had their own food supply. One mouse was sacrificed due to MRI evidence of intracranial bleeding after probe-implantation. No unexpected deaths occurred and all mice that died before study completion were euthanized based on one of the above criteria.

### PO_2_ Probe Implantation

**Fig 1:**
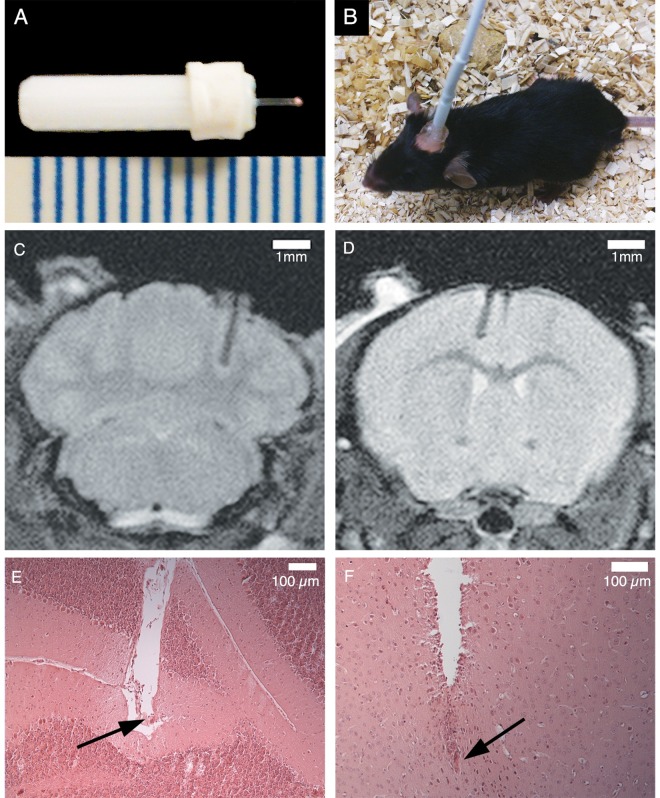
Implantation of fiber optic PO_2_ probe into mouse brain. **(A)** PO_2_ probe with scale in mm; the implanted fiber optics are to the right, with the platinum-based fluorophore embedded in the pink silicone tip. (**B)** An awake mouse during data collection, with fiber optic lead temporarily attached to the external end of the probe. **(C)** MRI (see Methods for imaging parameters) showing probe location after surgical implantation in the cerebellum and **(D)** the cortex. Grey matter has a higher pixel intensity than WM in this scan sequence. A typical cerebellar implant is shown in **(E)** at 10X magnification with H&E staining, showing the end of the fiber track directly in the granular layer of the cerebellar grey matter. There is little evidence of any inflammation (hypercellularity) occurring around the fiber. A typical cortical implant is shown in **(F)** (note the 90^0^ rotation relative to Fig 1D), again at 10X with H&E staining. The fiber tip was located within the grey matter of the cerebrum.

Tissue PO_2_ was measured with a fiber-optic oxygen sensor (Oxford Optronix, Oxford, UK) with an approximate diameter of 230 μm (Fig 1A) [29], implanted into the mouse cerebellum or cortex using stereotaxic methods [28]. Animals were anaesthetized with 5% isofluorane gas prior to surgery and spontaneously ventilated with 70% N_2_, 28% O_2_ and 2% isofluorane during surgery. Temperature and breathing rate were monitored throughout. Skin was folded back and a hole was drilled through the skull at stereotaxic coordinates (relative to bregma) +0.50 mm medial/lateral, -6.00 mm anterior/posterior for the cerebellum; +0.50 mm medial/lateral and +1.50 anterior/posterior for the cortex. The depth of the probe’s location was 2 mm below the top of the skull. The probe was secured with cyanoacrylate glue and a methacrylate skullcap. Fig 1B shows the completed skullcap with the probe tip protruding and a free-hanging optical fiber connected to an awake mouse. Mice were administered buprenorphine post-surgery for pain management and transferred to separate cages for recovery.

### Magnetic Resonance Imaging

Animals were scanned within 72 hours of implantation to verify probe position and rule out intracranial bleeding. MRI was done with a 9.4T MRI and a Bruker Avance console (Bruker Biospin GmbH, Rheinstetten, Germany). Animals were spontaneously ventilated with 70% N_2_, 28% O_2_ and 2% isofluorane; temperature and breathing rate were monitored throughout. Scans were performed using a rapid acquisition with relaxation enhancement (RARE) T_2_-weighted sequence: voxel resolution of 78 x 78 μm, slice thickness of 500 μm, matrix size of 256 x 256, TR = 3000 ms, TE = 16 ms, FOV = 20 mm and a RARE factor of 8 (Fig 1C and 1D).

### EAE Induction and Behavioral Scoring

EAE was induced using 50 μg of myelin oligodendrocyte glycoprotein (MOG) and 10mg/mL of heat-inactivated *Mycobacterium tuberculosis*, emulsified in complete Freund’s adjuvant (CFA) that was injected subcutaneously into the flanks as described previously [30]. An intraperitoneal injection of 300 ng of pertussis toxin (PTX) was given on the day of induction (day 0) and 2 days later. There were 8 EAE mice where the PO_2_ probe was in the cerebellum and 16 EAE mice where the PO_2_ probe was in the cortex. CFA controls were injected with everything above except MOG. There were 5 CFA controls with PO_2_ probes in the cerebellum and 8 CFA controls with PO_2_ probes in the cortex.

Symptom severity was measured with a 15-point grading scale [31], where the tail receives a maximum score of 2 for complete paralysis and each of the limbs receive a maximum of 3 for paralysis; a score of 15 indicates death. Scoring was done on the same day, one hour after PO_2_ measurement to minimize the effects of handling stress. The scorer was blinded to the experimental condition of the mouse to reduce bias.

### PO_2_ Measurement

During recording, an optical fiber was attached to the probe protruding from the methacrylate cap and the mouse was left free to move in its cage bottom. Tension was reduced on the head by supporting the fiber with an elevated stand. Individual animals were measured within 30 minutes of the same time of day. During measurement, light pulses are sent through the fiber to the platinum-based fluorophore, which fluoresces for a length of time that is inversely proportional to PO_2_ [29]. After a 15-minute acclimation period, PO_2_ data was recorded at 0.1Hz for 10 min (60 data points) per recording session. Descriptive statistics were calculated for each session.

### Data Analysis

In order to compare mice with greatly differing PO_2_ values, daily measurements were compared to an individual’s own baseline PO_2_ to determine the mean change in PO_2_ (ΔPO_2_). To determine hypoxia or hyperoxia, control measurements were grouped to create one dataset on each measurement day. Each EAE mouse measurement was then compared to the control dataset using a two sample t-test, with Bonferroni’s correction for repeated comparisons; significance was set at p < 0.05. If a ΔPO_2_ measurement was found to be significantly lower than controls it was labelled hypoxic, and if significantly higher it was labelled hyperoxic.

### Histology

Brain tissue was analyzed histologically in a subset of mice to determine if MRI localizations in GM were accurate. At sacrifice, animals were anaesthetized with an intraperitoneal injection of ketamine/xylazine, perfused through the heart with 10 mL of 1X PBS and then perfused once more with 10 mL of 10% neutral buffered formalin (NBF). Brain was excised and fixed in 10% NBF for at least 48 hours. Tissue was embedded in paraffin and sectioned at 8 μm, and subsequent staining was done with hematoxylin and eosin (H&E).

## Results

### Histology

PO_2_ probe implant site was measured with MRI in all animals, but the actual location was confirmed histologically in a subset of mice (4 cerebellum and 5 cortex implants). This provided confidence that the MRI localization in GM was accurate. All probe tips were located in the GM. Representative sections for the cerebellum and cortex are shown in Fig 1E and 1F, respectively.

### Absolute Cerebellar PO_2_ Measurements

**Fig 2:**
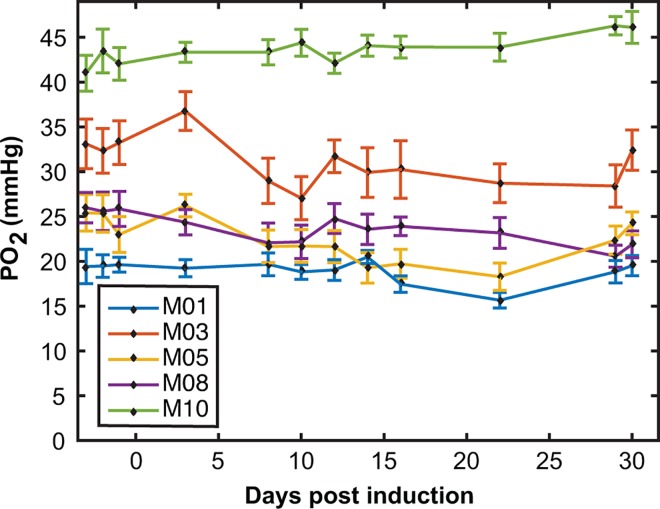
Heterogeneity of absolute PO_2_ measurements. Raw PO_2_ data collected from the cerebellum for 5 CFA mice, plotted over the entire disease course. Diamonds and error bars show PO_2_ mean ± SD on a measurement day. The mean baseline values for the cerebellum CFA mice range from 19.5±1.4 to 42.2±2.3 mmHg (mean ± SD). This heterogeneity in absolute PO_2_ values necessitated the use of ΔPO_2_ from baseline in order to make comparisons between mice.

Absolute PO_2_ values are shown over the disease time-course in Fig 2 for cerebellum control mice. Pre-induction (days -3 to -1) mean ± SD PO_2_ values for the CFA mice were 19.5±1.4, 32.9±2.6, 24.6±2.3, 25.8±1.9 and 42.2±2.3 mmHg. This shows the high between-animal heterogeneity of the data, supporting the argument for analyzing the data using ΔPO_2_ from baseline.

The pre-induction PO_2_ values for all the animals combined was 29.9±6.8 mmHg (mean ± SD, n = 13) for the cerebellum and 23.6±4.8 mmHg (n = 24) for the cortex. The cerebellar PO_2_ was significantly higher than the cortex (t-test, p = 0.002).

### Cerebellum

**Fig 3:**
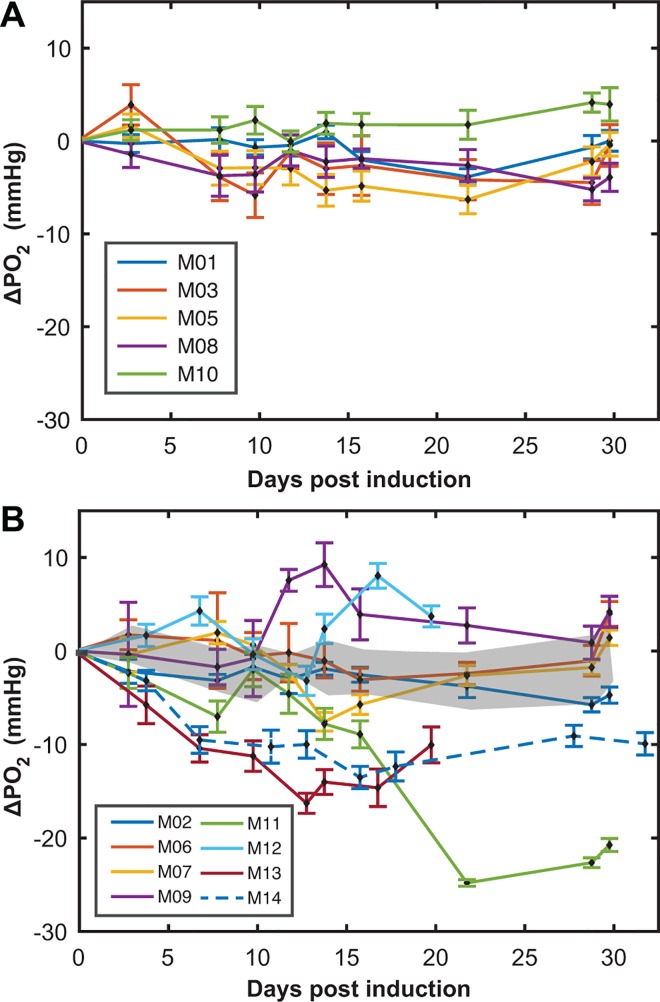
Measurements of cerebellar PO_2_ over disease time-course. Changes in PO_2_ from baseline for (**A**) CFA controls (n = 5) and (**B**) EAE mice (n = 8) over the time-course of the disease. PO_2_ changes were calculated by subtracting an animal’s baseline value (the average of the 3 days before induction) from their daily measurement. The shaded region in (B) represents the mean ± SD of ΔPO_2_ from the grouped CFA dataset shown in (A). (mean ± SD)

Measurements of cerebellar oxygenation over the full disease time-course are shown as ΔPO_2_ from baseline in Fig 3A & 3B for CFA and EAE mice, respectively. Oxygenation remained relatively stable throughout the time-course in controls (Fig 3A). However, the EAE population showed an increase in variance by the first time point (Fig 3B), and reached a maximum variance between 10 and 20 days post-induction. Furthermore, both hypoxia and hyperoxia were observed: 7 of 8 EAE mice became hypoxic and 4 of 8 EAE mice became hyperoxic on at least 1 day (Table 1). There was a total of 34/67 (51%) hypoxic days and 18/67 (27%) hyperoxic days, indicating a much greater frequency of hypoxic events than hyperoxic events.

**Table 1:** Summary data for cerebellar EAE mice.

Mouse	# of Hypoxic Days	# of Hyperoxic Days	Average ΔPO_2_ for hypoxia (mmHg)	Average ΔPO_2_ for hyperoxia (mmHg)	Day with largest absolute ΔPO_2_ (mmHg)	Peak behavior	Day of peak behavior
M02	5	0	-3.8 ± 1.4	N/A	29	0	N/A
M06	1	3	-3.1 ± 1.2	1.8 ± 2.3	30	7	22
M07	3	3	-4.0± 3.3	1.3 ± 0.7	14	4	16
M09	0	7	N/A	4.0 ± 3.4	14	6	14
M11	8	0	-12.3 ± 8.9	N/A	22	4	16
M12	1	6	-3.2 ± 1.6	3.3 ± 2.8	17	7.5	20
M13	7	0	-11.8 ± 3.5	N/A	13	11	20
M14	8	0	-9.7 ± 3.0	N/A	14	8.5	16

A measurement day was considered hypoxic if ΔPO_2_ was significantly lower than the grouped ΔPO_2_ from CFA controls on the same day and hyperoxic if significantly higher (2-sample t-test with Bonferroni’s correction). In total, there were 34/67 hypoxic days and 18/67 hyperoxic days. Hypoxic days were also significantly more extreme with an overall average ΔPO_2_ of -8.8 mmHg, for hypoxic days vs. +3.2 mmHg for hyperoxic days. Significance was determined by comparing the absolute values of ΔPO_2_ hypoxic and hyperoxic days with a 2-sample t-test, p < 0.001.

Hypoxic days showed larger changes from baseline, with a mean value ΔPO_2_ of -8.8±6.0 mmHg vs. +3.2±2.8 mmHg for hyperoxic days. The absolute values of these data were significantly different using a 2-sample t-test, p < 0.001.

**Fig 4:**
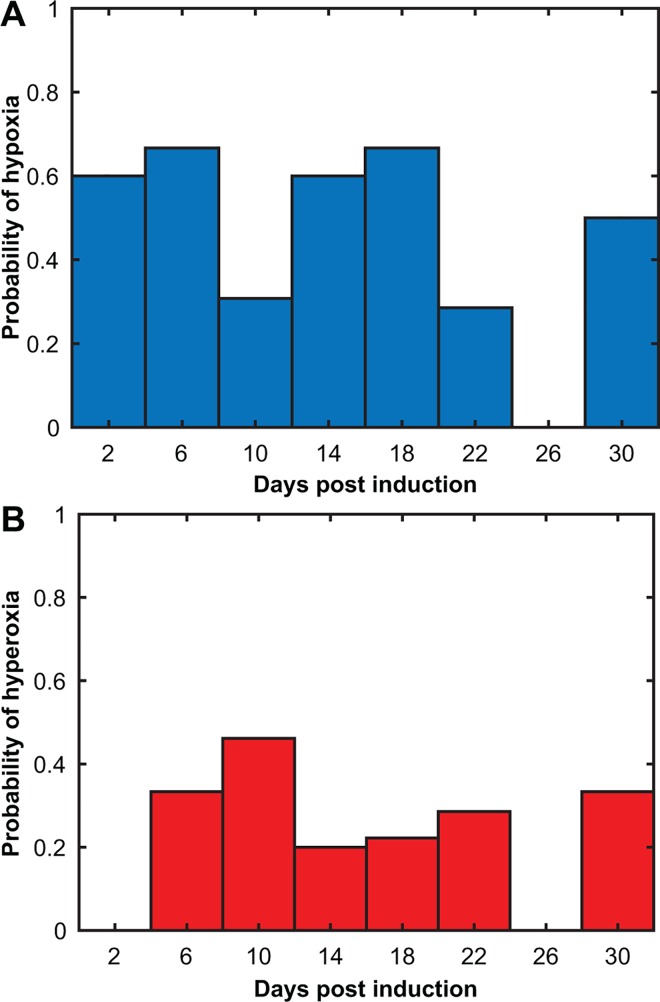
Probability of cerebellar hypoxia and hyperoxia over disease time-course. Probability of hypoxia (A) and hyperoxia (B), calculated by dividing the total number of significant hypoxic (or hyperoxic) days by the total measurements. There is a higher probability of hypoxia than hyperoxia.

The probability of hypoxia or hyperoxia being measured in the cerebellum EAE dataset is shown in Fig 4A and 4B, respectively. The probabilities were determined by counting the number of hypoxic or hyperoxic measurements in a time range and dividing by the total number of measurements in that same time period. The period of the highest probability of detecting hypoxia occurs at days 4–8 post-induction, with a value of 0.67, although there is a high probability during peak score times as well. For hyperoxia, the maximum occurs at days 8–12, with a value of 0.46. A high probability of hypoxia occurs before onset of symptoms.

### Behavior and Cerebellar PO_2_

**Fig 5:**
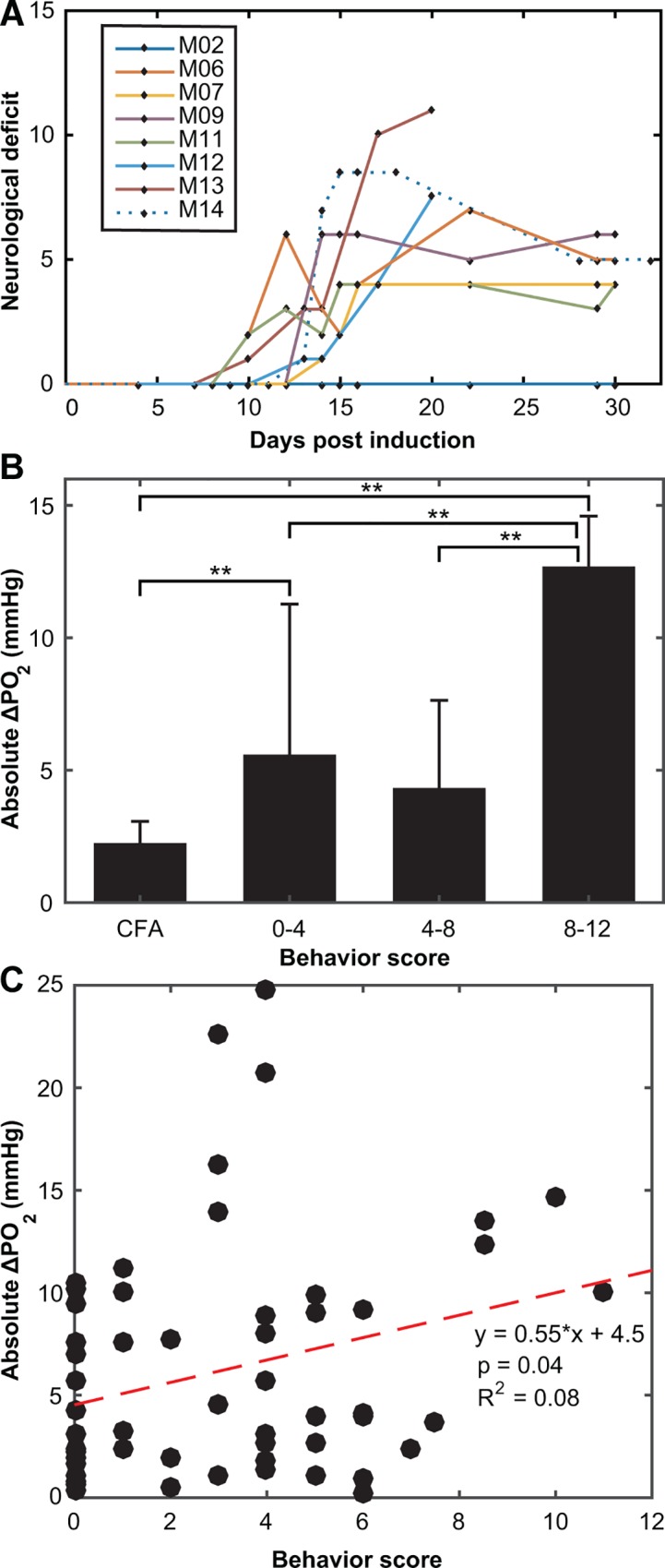
Behavioral deficits correlate with increasingly abnormal oxygen measurements in cerebellar gray matter. **(A)** Behavior deficit scores are shown over the course of the disease. A 15-point scale was used (see Methods) with the scorer blinded to mouse’s disease status and oxygen measurements. **(B)** Mean ± SD of absolute ΔPO_2_ (including all measurement days) grouped by behavior score. ΔPO_2_ absolute values increase as behavioral deficit increases. **: one-way ANOVA, p<0.05. (**C**) Oxygenation is disrupted in symptomatic EAE mice and this disruption increases with behavioral deficit. A weak correlation was found (R^2^ = 0.08; p = 0.04) between behavioral score and absolute ΔPO_2_ for mice with a maximum behavior score > 0.

Behavioral deficits were first seen at day 10 (Fig 5A), with behavioral deficits in symptomatic mice persisting until sacrifice. 6/8 mice had a score greater than 4, indicating complete tail paralysis and some hind limb paralysis. 1/8 had a score greater than 10, considered to be severely paralyzed. One mouse had a behavior score of zero for the entire experiment.

Significant hypoxia was seen in the cerebellum before the onset of clinical symptoms (day 4 for PO_2_ vs. day 10 for clinical symptoms). The ΔPO_2_ magnitude increased with increasing behavioral deficit scores when grouped into low (0–3.9), medium (4–7.9) and high deficit score (8.0–12) (Fig 5B). The low and high groups had significantly higher absolute ΔPO_2_ compared to controls (one way ANOVA in SPSS with a Games-Howell post-hoc test to account for unequal variances, p < 0.05). In addition, regression analysis was performed on the dataset (Fig 5C), with a weak correlation (R^2^ = 0.08) found between behavioral deficit and absolute ΔPO_2_ in mice that showed behavioral symptoms. The linear model was tested against the null hypothesis of y = 0 using an F-test (F = 4.59) and found to be significantly different, p = 0.04. Absolute PO_2_ measurements for EAE mice did not differ when grouped by behavioral score.

### Cortex

**Fig 6:**
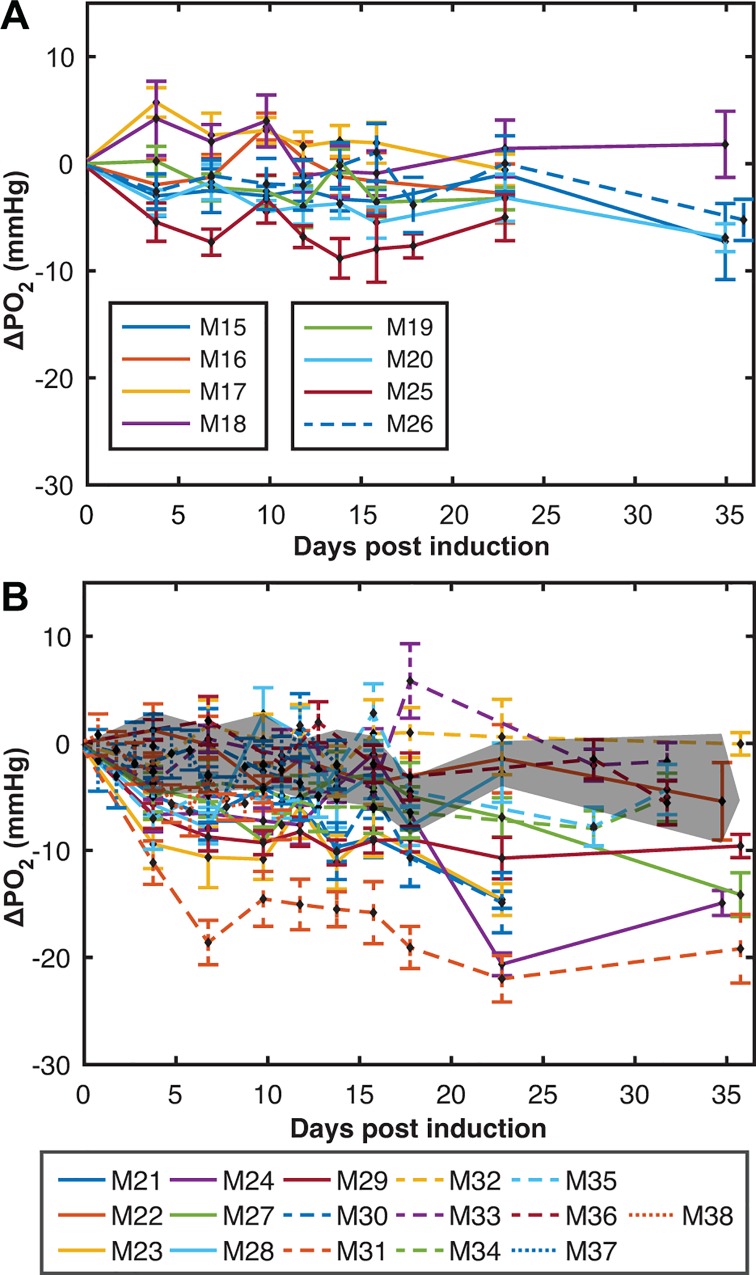
Measurements of cortical PO_2_ over disease time-course. ΔPO_2_ from (**A**) CFA controls (n = 8) and (**B**) EAE mice (n = 16) up to 36 days after EAE induction. ΔPO_2_ was calculated by comparing an animal’s daily measurements to their own baseline measurements. The shaded region in (B) represents the mean **±** SD of ΔPO_2_ from the grouped CFA dataset. (mean ± SD)

The change in PO_2_ in the cortex shows a similar pattern as that in the cerebellum, yet with more pronounced hypoxia. Fig 6A and 6B show the cortical PO_2_ measurements for CFA and EAE mice, respectively. ΔPO_2_ of controls remained stable throughout the experiment (Fig 6A). Qualitatively, an increase in the variance of the EAE population was seen at the first time point (day 3–4) and remained high through the study (Fig 6B). Table 2 summarizes the analysis on the cortical EAE mice. 14/14 mice had at least one hypoxic day and 9/14 had at least one hyperoxic day. 82/112 days were found to be hypoxic when compared to the control dataset and 22/112 were found to be hyperoxic, indicating that hypoxia is the dominant state of oxygenation in the cortex. Again, significance of hypoxia or hyperoxia was determined by a 2-sample t-test with Bonferroni’s correction, with a significance threshold of p < 0.05. Hypoxic days were more severe than hyperoxic days, with mean ΔPO_2_ values of -8.0±4.6 mmHg and +0.8±2.1 mmHg, respectively. The absolute values of these were found to be significantly different using a 2-sample t-test, p < 0.001.

**Table 2:** Summary data for cortex EAE mice.

Mouse	# of Hypoxic Days	# of Hyperoxic Days	Average ΔPO_2_ for hypoxia (mmHg)	Average ΔPO_2_ for hyperoxia (mmHg)	Day with largest absolute ΔPO_2_ (mmHg)	Peak behavior	Day of peak behavior
M21	7	0	-7.9±3.6	N/A	23	8.5	23
M22	1	2	-4.1±2.3	0.5±0.9	35	2	14
M23	7	0	-10.1±2.7	N/A	23	2	14
M24	7	1	-9.7±5.9	-1.0±2.4	23	4	16
M27	7	0	-6.9±3.7	N/A	36	3.5	23
M28	4	2	-6.6±1.4	1.8±1.3	7	11.5	14
M29	9	0	-9.1±1.1	N/A	23	4	18
M30	5	0	-8.6±5.0	N/A	23	10	16
M31	9	0	-16.8±3.2	N/A	23	6.5	23
M32	1	6	-2.2±2.5	0.9±0.6	14	4.5	18
M33	2	3	-4.0±0.3	1.5±3.9	18	9.5	18
M34	6	0	-5.5±1.5	N/A	28	9	18
M35	5	2	-5.9±1.4	-0.8±5.2	28	6	32
M36	1	4	-1.9±1.5	0.6±2.4	32	5	32
M37	6	1	-3.3±1.4	1.7±3.0	8	12	19
M38	8	1	-5.1±1.7	0.8±1.9	11	0	N/A

A measurement day was considered hypoxic if it was found to be significantly lower than the grouped ΔPO_2_ measurements from CFA controls on the same day. A measurement was considered hyperoxic if it was found to be significantly higher. In total, there were 85 hypoxic days and 22 hyperoxic days. Hypoxic days were also significantly more extreme, with an overall average ΔPO_2_ of -8.0 ± 4.6 mmHg for hypoxic days vs. +0.8 ± 2.1 mmHg for hyperoxic days.

**Fig 7:**
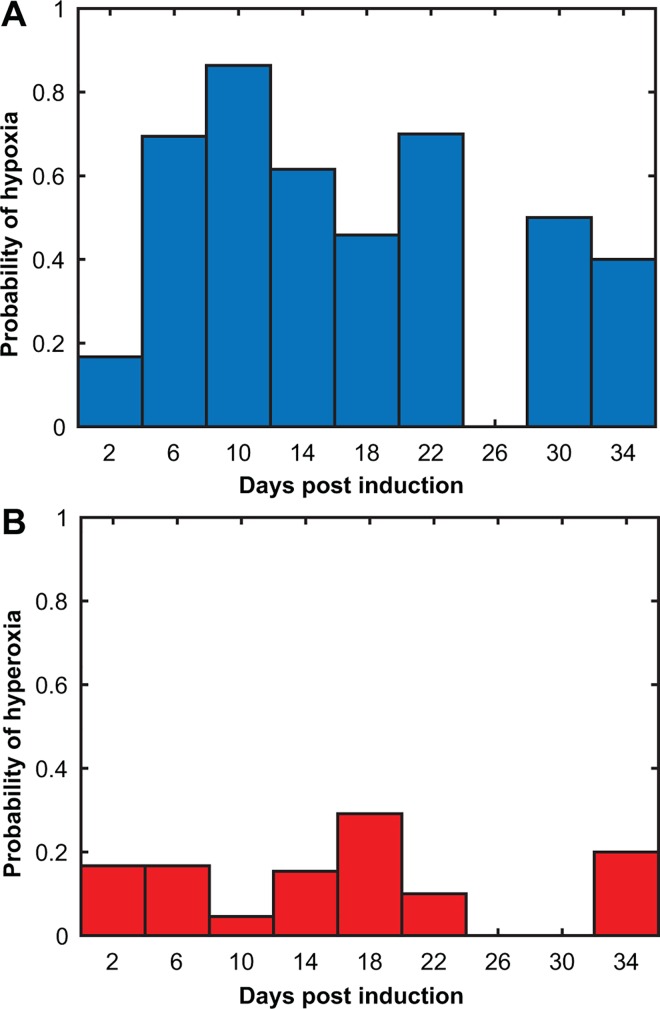
Probability of cortical hypoxia and hyperoxia over disease time-course. Probability of hypoxia (A) and hyperoxia (B). The period with the highest probability of hypoxia in the cortex was between days 8–12 and for hyperoxia was between days 16–20.

The probability of hypoxia or hyperoxia being measured as a function of time for the cortical EAE dataset is shown in Fig 7A and 7B, respectively. As in Fig 4, the probabilities were determined by counting the number of hypoxic or hyperoxic measurements in a time range and dividing by the total number of measurements in that same time period. The maximum probability of detecting hypoxia occurs at days 8–12 post-induction with a value of 0.86 and extends through the peak period. For hyperoxia, the maximum occurs at days 16–20, with a value of 0.29. Note that the peak hypoxia probability occurs after the peak for the cerebellum and coincides with the beginning of behavioral deficits.

### Behavior and Cortical PO_2_

**Fig 8:**
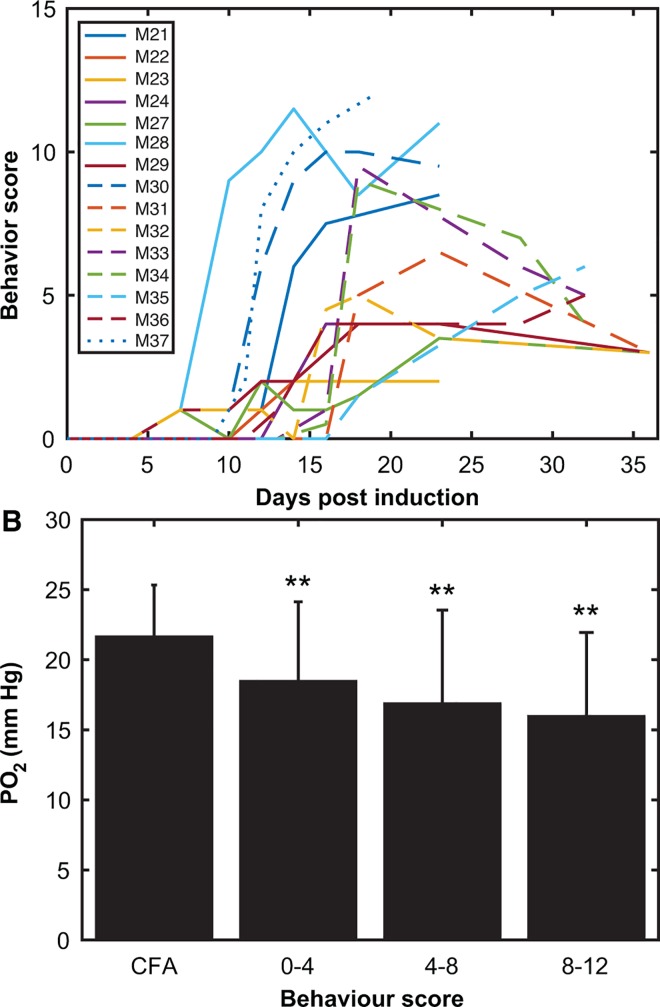
Behavioral deficits correlate with decreasing PO_2_ in cortical grey matter. **(A)** Behavior deficit scores are shown over the course of the disease. Deficits were seen by day 7. 10/16 mice had a score > 4 and 3/16 had a score > 10. One mouse was not included as it never showed symptoms. **(B)** Absolute PO_2_ values grouped by behavior score. PO_2_ values decrease as behavioral deficit increases (t-test with Bonferroni’s correction; mean ± SD, ** = p < 0.01).

Behavioral deficits in cortex mice were seen as early as day 7 (Fig 8A) and persisted in all mice until time of sacrifice. 10/16 mice had a maximum score greater than 4 (indicating complete tail paralysis and some hind limb paralysis) and 3 mice had a score greater than 10 **(**considered to be severely paralyzed). A significant decrease in absolute PO_2_ was found with increasing behavior scores (Fig 8B). 2-sample t-tests were performed between each behavior group and CFA controls, with Bonferroni’s correction. All groups were significantly lower than controls with p < 0.01.

## Discussion

### The measurement of gray matter PO_2_

In this study we performed *in vivo* tissue oxygenation measurements in the cerebellum and cortex of awake, unrestrained mice for the first time. As this is a novel procedure, we outline below some of the strengths and weaknesses of this technique.

First, the ability to measure oxygenation in awake mice eliminates the confounding effects of anesthesia. In addition, we took steps to minimize stress levels in the mice by exposing them to human contact and the measurement procedure, before the initial baseline measurements, and by ensuring the measurements took place in a quiet room. This acclimation period also allowed time for the brain tissue to recover after the implantation procedure.

PO_2_ was detected through the increased quenching of fluorescent emission by a platinum-based fluorophore. Since the characteristic shape of this decay curve will not change over repeated use, calibration of the probes remains stable. However, the data are less variable at lower PO_2_ levels [29]. In addition, the probes sample an approximate volume of 0.5–1 mm^3^, which would mean any large-scale spatial heterogeneity in PO_2_ was not captured.

The probes measure with a high sampling rate, allowing for measurement of high-frequency oscillations in PO_2_. However, this also means that each daily PO_2_ measurement contains a large number of data points, making it more likely that one will see differences between daily measurements. When we used ANOVA to determine if PO_2_ values in a given animal changed relative to pre-induction values, we found that controls as well as EAE animals showed differences on nearly every day for every animal. Also, the between animal differences in PO_2_ were often large compared to the changes we were expected with EAE induction. For these reasons, we developed a protocol for determining differences that used ΔPO_2_.

### Oxygenation and hypoxia in GM

We define hypoxia as a PO_2_ value that is lower than ‘normal’ [32]. In our study, we define normal as the PO_2_ in the CFA controls (animals that had all immune stimulation but no sensitization to MOG or WM). We did this by calculating the changes in PO_2_ from a pre-induction baseline. During the time-course, the changes in the controls for a given day were compared with the changes in the individual EAE mice.

The data clearly show that there is increased variability in PO_2_ in EAE mice. Using our definition, we also show that hypoxia is more frequent than hyperoxia. The presence of hypoxia in approximately 87% or more of the EAE mice (in cortex and cerebellum) supports our hypothesis that there is significant hypoxia in GM in the EAE mouse.

Hypoxia-like lesions were observed in MS patients [21], where HIF-1α levels were elevated along with downstream genes like vascular endothelial growth factor (VEGF) [23]. Increased levels of ER-stress molecules and molecules associated with hypoxia were seen in GM lesions of MS patients [20, 33].

Previous work also indicates that there is diffuse GM hypoxia in MS, not necessarily associated with lesions. Using quantitative near-infrared spectroscopy, it has been reported that approximately half of MS patients have reduced oxygen saturation in cortical GM [24]. Reduced oxygen saturation is likely to correlate with reduced tissue PO_2_. In the EAE mouse spinal cord, hypointense regions were detected using susceptibility-weighted MRI, indicating elevated deoxyhemoglobin levels [25]. This is likely to occur if regional microvascular oxygen saturation is reduced [25]. This also supports the work of Davies *et al*., who reported hypoxia in the spinal cord of anaesthetized EAE rats [26].

The probability of hypoxia as a function of time (see Figs 4 and 7) would indicate that hypoxia occurs earlier in the cerebellum than the cortex. It has been suggested that EAE pathology starts at the caudal end of the spinal cord and moves in a rostral direction; our data supports this [34]. Significant WM loss occurs by peak disease in the cord [35] and global GM degeneration has been reported to occur in the cerebellum, cortex and whole brain in EAE mice by 80 days post-induction [14]. Thus, cortical involvement may require longer times post-induction than in the spinal cord or the cerebellum. Interestingly, this could also mean that the EAE is a good model of progression, as there is evidence of long term cortical GM pathophysiology.

There is some indication that hypoxia in cortex relates to the level of behavioral impairment (Fig 8B). A decline in PO_2_ in the spinal cord was reported to correlate with increased severity of symptoms in a rat EAE model [26]. However, in the cerebellum we did not find a significant relationship between hypoxia and symptoms, although there was a trend. Instead, there was a relationship between the absolute PO_2_ change (in either direction) (Fig 5), indicating that variance in PO_2_ also indicates pathology of some degree.

### Potential cause of hypoxia

A hypoxic cellular environment arises when there is an undersupply of oxygen relative to the metabolic needs in the region. As the EAE mouse is an inflammatory model, it is possible that the inflammation itself is part of the mechanism causing hypoxia. In a rat EAE model, it has been suggested that hypoxia may relate to the level of inflammatory cell infiltration [26]. Inflammatory cells could impair microvascular flow by direct blockage or by impairing vascular regulation (and neurovascular coupling), possibly by an increase in reactive oxygen species damaging the vascular endothelium. Blood vessel damage is often seen in MS lesions [36].

Changes in metabolism could also affect oxygenation, since cells with lower metabolism require lower oxygen gradients for their mitochondria to function properly and vice versa for higher metabolism [37]. With normal neurovascular coupling, increased metabolism correlates with higher PO_2_ and thus, decreased metabolism is likely to correlate with reduced PO_2_ [37, 38].

Multiple lines of evidence indicate that there is reduced metabolism in MS. Resting state metabolism, as measured with ^18^F-fluorodeoxyglucose PET imaging, is reduced in many cortical and subcortical GM regions [39–41]. Reductions in cerebral metabolic rate of oxygen (CMRO_2_) have been reported in MS patients [17, 42, 43] and reduced metabolic rate has been correlated with cortical degeneration and cognitive disability in progressive stage patients [17]. There is also evidence for reduced GM perfusion in MS patients [44–46], indicating reduced metabolism that correlates with cognitive deficits [45].

Hyperoxia in a smaller subset of mice is harder to explain. If neurovascular coupling is indeed impaired, then decreases in metabolism may not be accompanied by an appropriate reduction in perfusion—resulting in elevated PO_2_. Alternately, there may be regions of high metabolic rate caused by factors such as increased inflammation, or the fact that demyelinated axons require more energy to generate action potentials.

### Implications of hypoxia—the hypoxia/inflammation cycle

We would argue that the decline in PO_2_ we report is significant when it comes to the regulation of the hypoxia response. HIF-1α is a master regulator of this process. In normoxic conditions, prolyl hydroxylase (PHD) hydroxylates HIF-1α, which targets it for degradation. In hypoxia, PHDs are inhibited allowing the HIF-1α subunit to increase in concentration [47, 48], bind to HIF-1β, transfer to the nucleus and trigger hypoxia-related gene transcription. Genes include those for angiogenesis and glycolytic activity [49, 50].

The K_m_ for PHD1-3 is reported to be in the range of 85–240 μM [51, 52]. Our measurements of neurons were between 33–75 μM (20–45 mmHg, adjusted for an altitude of 1100m) at baseline. This puts neurons very much in the sensitive regime of the hydroxylase’s kinetics when at physiological dissolved oxygen levels, thus ensuring that cells will be able to efficiently respond to small or large decreases in oxygen.

As a typical example, a drop of 8.8mmHg (the average reduction during hypoxic days) from the mean cerebellar baseline PO_2_ of 29.3 mmHg down to 21.1 mmHg corresponds to a decline of 41.6 μM to 29.4 μM. Using the Michaelis-Menton equation [53], this results in a drop of 22–26% in the initial reaction rate of the PHD-O_2_ complex, depending on the K_m_ value used. This change is enough to impact flux rates in this metabolic pathway.

There is an interaction between hypoxia and inflammation, which is particularly relevant given that inflammation is a factor in MS and we have detected hypoxia. NF-κB is a master regulator of the inflammatory response [54], which stimulates the expression of pro-inflammatory cytokines such as TNF-α, IL-1β and IL-69, iNOS [55] and adhesion molecules [56]. NF-κB also upregulates HIF-1α [57]. Furthermore, NO and TNFα activated macrophages, components of the inflammatory response, result in increased HIF-1α [58].

Hypoxic inhibition of PHDs leads to upregulation of HIF-1α, but also leads to activation of NF-κB, triggering an immune response [59]. HIF-1α also stimulates aggregation and invasiveness of myeloid cells [60]. Inflammation can even trigger a hypoxia response in normoxia with NO inhibition of PHD’s [61][58].

It may be that an initial inflammatory event triggers a hypoxia/inflammation cycle in the micro-environment and that breaking this cycle will create a more favorable environment for remyelination and reduce atrophy. The regulation of these two pathways are linked. Understanding this linkage will be key to understanding the role of hypoxia in CNS inflammation.

## Conclusions

We have demonstrated, for the first time, that one can use the implanted, fibre optic based PO_2_ sensors to measure brain oxygenation in awake, unrestrained mice over time. This is also the first time that measurements of PO_2_ have been obtained in the brain of an animal model of MS or in the cerebellum of any animal. We demonstrated that there is increased variance in oxygenation after induction of autoimmunity. Although hyperoxia was periodically observed, the predominant change was to a hypoxic condition. There was significant hypoxia in GM of both the cerebellum and cortex, with cortical hypoxia occurring in approximately 75% of the measurements. The average decline in PO_2_ was -8.8±6.0 mmHg (mean ± SD) for the cerebellum and -8.0±4.6 for the cortex, which we argue is sufficient to stimulate a hypoxia response and to possibly modulate the immune response. There is some indication that behavioral impairment becomes greater with increased hypoxia and variance in oxygenation. The cerebellum appears to become hypoxic first, supporting the suggestion that the involvement in this EAE model moves in a rostral direction from spinal cord to cortex. Both hypoxia and increased PO_2_ variance are indicative of an abnormal balance between oxygen delivery and utilization, supporting the argument that there is significant GM pathophysiology. This hypoxia could increase the severity of neurodegeneration, inhibit remyelination and relate to inflammation.
